# *Leishmania infantum* Virulence Factor A2 Protein: Linear B-Cell Epitope Mapping and Identification of Three Main Linear B-Cell Epitopes in Vaccinated and Naturally Infected Dogs

**DOI:** 10.3389/fimmu.2018.01690

**Published:** 2018-07-25

**Authors:** Monique Paiva Campos, Fabiano Borges Figueiredo, Fernanda Nazaré Morgado, Alinne Rangel dos Santos Renzetti, Sara Maria Marques de Souza, Sandro Antônio Pereira, Rodrigo Nunes Rodrigues-Da-Silva, Josué Da Costa Lima-Junior, Paula Mello De Luca

**Affiliations:** ^1^Laboratório de Pesquisa Clínica em Dermatozoonoses em Animais Domésticos, National Institute of Infectology Evandro Chagas-Fiocruz, Rio de Janeiro, Brazil; ^2^National Institute of Infectology Evandro Chagas-Fiocruz, Rio de Janeiro, Brazil; ^3^Instituto Carlos Chagas, Fundação Oswaldo Cruz, Curitiba, Brazil; ^4^Laboratório de Pesquisa em Leishmanioses, Instituto Oswaldo Cruz, Fundação Oswaldo Cruz, Rio de Janeiro, Brazil; ^5^Laboratório de Imunoparasitologia, Instituto Oswaldo Cruz, Fundação Oswaldo Cruz, Rio de Janeiro, Brazil

**Keywords:** canine visceral leishmaniasis, vaccines, A2 protein, serology test, epitope mapping, epitope prediction

## Abstract

In Brazil, canine visceral leishmaniasis (CVL) is caused by *Leishmania infantum*, presenting a broad spectrum of clinical manifestations. Dogs are the main parasite reservoir in urban areas and canine cases precede human infection. Currently, A2 protein based Leish-Tec® vaccine is the only vaccine commercially available against CVL in Brazil. Considering that the main screening and confirmatory tests of canine infection are serological, it is possible that the antibody response elicited after vaccination interfere with diagnosis, leading to the inability to distinguish between vaccinated and infected animals. In order to identify the specific B-cell response induced after vaccination, A2 protein sequence was screened for main linear B-cell epitopes using *in silico* prediction (Bepipred) and immunological confirmation by ELISA. Three amino acid sequences were described as potential B-cell epitopes (SV11-SAEPHKAAVDV, PP16-PQSVGPLSVGPQSVGP, and VQ34-VGPLSVGPQSVGPLSVGPLSVGPQAVGPLSVGPQ). Specific IgG ELISAs were performed in sera of 12 immunized dogs living in non-endemic areas, followed for up to 1 year after immunization. The results were compared with those obtained in a group of 10 symptomatic and 10 asymptomatic CVL dogs. All predicted epitopes were confirmed as linear B-cell epitopes broadly recognized by sera from studied dogs. Total IgG ELISAs demonstrated distinct patterns of response between peptides in the immunized and CVL groups. VQ34 peptide was recognized by the majority of sera from vaccinated and symptomatic dogs, and increases after vaccination. PP16 induced low levels of specific IgG that increased 1 year after immunization. Interestingly, a low frequency of reactivity was found against SV11 in naturally infected dogs (symptomatic and asymptomatic), while 83.3% of vaccinated dogs presented positive responses 1 year after immunization. The two animals in the vaccinated group that did not respond to SV11 1 year after immunization presented positive serology both 30 days and 6 months after immunization. In summary, we identified three main linear B-cell epitopes in A2 based vaccine. Moreover, the humoral response against SV11 presented marked differences between infected and Leish-Tec vaccinated dogs, and should be further investigated, in large trials, to confirm its potential as a serological marker able to distinguish between infected and vaccinated dogs.

## Introduction

Visceral leishmaniasis (VL) is a vector-borne disease transmitted by female sandflies. In the American continent is a zoonotic disease caused by *Leishmania infantum* (1). *Lutzomyia longipalpis* is the most important vector and the dog (*Canis familiaris*) the main reservoir in the domestic and peridomestic cycle (2). As a prophylactic practice to control VL, World Health Organization recommends the systematic treatment of human cases, vector control, and elimination of the domestic reservoir, mainly seropositive-infected dogs (3). In Brazil, one of the main attempts to control VL is the identification and euthanasia of seropositive dogs in endemic areas (4). Unfortunately, this measure, besides causing social discomfort, does not seem to be effective, since the disease is expanding to non-endemic areas (5). Mathematical modeling suggests that this approach fail because of the high incidence of canine infection and infectiousness, the insensitivity of the diagnostic tests to detect infectious dogs, and time delays between diagnosis and culling observed in public health services (6). Moreover, Dye suggested that the control of phlebotomine population allied to dogs’s vaccination would be more effective in controlling VL than dog culling (7).

Currently in Brazil, only Leish-Tec^®^ vaccine (Hertape Calier Animal Health^®^, Minas Gerais, Brazil) has the license for commercialization granted by the Brazilian Ministry of Agriculture, Livestock and Supply (MAPA). On the other hand, despite some promising results, the Brazilian Ministry of Health (BMH) does not recommend its use as a form of VL control, since studies related to this approach would still be preliminary (8). One of the reasons for this non-recommendation would be the possible interference of the humoral response induced by the vaccine in the serological tests recommended by the BMH to CVL diagnose, which could lead to an impossibility of distinguishing vaccinated from infected animals.

The current protocol for serological diagnosis adopted by the BMH uses a rapid dual-path platform test (TR DPP^®^) for screening, followed by enzyme-linked immunosorbent assay (ELISA) for confirmation, and DPP-negative animals are considered free of infection (9). Although the new protocol has brought a significant improvement in the diagnostics of CVL (10), the specificity of both TR DPP^®^ and ELISA can be low, and more efficient tests must be identified and combined with others for the improved identification of naturally infected dogs (11–15).

The main component of Leish-Tec^®^ vaccine is the *Leishmania donovani* amastigote stress response protein A2, required for parasite survival in visceral organs in mice (16, 17), and involved with the induction of a potent CVL protective type 1 cellular immune response (18). Since A2 protein is not expressed in the promastigote form, its use as a vaccine and in a diagnostic test was suggested (19). Ghedin et al. (19) also shown that 82% of sera of patients with kala-azar from Sudan present positive ELISAs to recombinant A2 protein (rA2). Likewise, Carvalho et al. (20) suggested A2 protein as a potential tool for the diagnosis of VL and CVL, since 77% sera from symptomatic VL patients presented positive ELISAs to rA2. The results obtained with symptomatic and asymptomatic dogs were more promising, showing a positivity of 93.3 and 77%, respectively, for each group in the rA2 ELISA. It was further demonstrated that rA2 ELISA is more sensitive for asymptomatic dogs (88%) than rK39 and rK26 and also poses the greatest specificity (98%) (21).

Considering that the main screening and confirmatory tests of canine infection in Brazil are serological, it is possible that the antibody responses elicited by the vaccine interfere with the diagnosis, leading to the inability to distinguish between vaccinated and naturally infected animals. We have recently demonstrated that in a non-endemic area, and up to 1 year after Leish-Tec^®^ immunization, vaccinated dogs remained negative in the DPP/EIE serological protocol used by the BMH (22, 23). On the other hand, the high degree of serological response in rA2 ELISAs observed in previous studies with sera of CVL animals (19–21) led us to evaluate the potential role of this protein for diagnosis, focusing on possible differences in the responses elicited against immunodominant linear B cell epitopes in a group of Leish-Tec^®^ vaccinated dogs and in two groups of CVL animals (symptomatic and asymptomatic), naturally infected by *L. infantum*.

Therefore, aiming to identify epitopes with potential to distinguish between vaccinated dogs and dogs naturally infected by *L. infantum*, the A2 protein sequence [GenBank: GQ290460] was screened for main linear B-cell epitopes using *in silico* prediction (Bepipred) followed by immunological confirmation by ELISA.

## Materials and Methods

### Ethical Statement

The experimental protocols of this study were approved by the Ethical Committee for the Use of Experimental Animals (CEUA) of Fundação Oswaldo Cruz—FIOCRUZ (naturally infected animals—Protocol LW-4/17) and of Instituto Oswaldo Cruz—IOC/FIOCRUZ (healthy and vaccinated animals—Protocol L-45/2015).

### Dogs

Serum of 12 healthy dogs living in non-endemic areas for CVL and from 20 dogs naturally infected with *L. infantum* were used in the study.

The healthy dogs were all of known history and defined breeds (Table 1), dwell in individual pens, and received balanced feeding. In addition, they are regularly vermifuged and vaccinated against the most common canine infections.

**Table 1:** Dog characterization accordingly to gender, weight, age, breed, clinical classification, serological response to *Leishmania*, parasite isolation, and detection by quantitative polymerase chain reaction (qPCR).

Dogs	Gender	Weight (kg)	Age (months)	Breeds	Clinical classification (score)	Serology (DPP-LVC)^®^	*Leishmania infantum* isolation + qPCR [bone marrow (BM)]
Leish-Tec^®^ (*n* = 12)	6 males	18–50	11–66	4 Shepherd Malinois	Low	T0 = 0/12	T0 = 0/12
	6 females			3 German Shepherds	(0) *n* = 12	T1 = 0/12	
				4 Rottweilers		T6 = 1/12^a^	
				1 Labrador		T12 = 0/12	

CVL (*n* = 10)	7 males	5–15	24–192	8 mixed breeds	Low	**Serology (DPP-LVC^®^ and EIE-CL)**	10/10
	3 females			1 Poodle	(1) *n* = 1	10/10	
				1 Dachshund	Moderate		
					(4) *n* = 1		
					High		
					(7) *n* = 2		
					(8) *n* = 1		
					(9) *n* = 4		
					(11) *n* = 1		

ACVL (*n* = 10)	7 males	5–25	12–84	9 mixed breeds	Low	10/10	9/10
	3 females			1 Labrador	(0) *n* = 9		
					(1) *n* = 1		

*^a^One dog immunized with Leish-Tec^®^ presented positive DPP-LVC^®^ 6 months after vaccination although the EIE test was negative. Parasitological culture and qPCR were performed with BM and blood samples collected at the same time point and the results were negative. 1 year after immunization (T12), this dog tested negative in the DPP-LVC^®^. All dogs from CVL and ACVL groups presented positive DPP-LVC^®^ and EIE tests*.

*Leish-Tec^®^—vaccinated dogs, CVL – canine visceral leishmaniasis, ACVL – asymptomatic canine visceral leishmaniasis*.

After clinical examination and negative serological and parasitological tests (Table 1), these dogs were immunized with the Leish-Tec^®^ vaccine (Lot# 034/14—Hertape Calier Saúde Animal^®^, Minas Gerais, Brazil) and followed for up to 1 year. Blood samples were taken to obtain the serum at the time of the first evaluation (before vaccination T0) and 1 month (T1), 6 months (T6), and 12 months (T12) after the ending of immunization protocol recommended by the manufacturer (three doses with an interval of 21 days in dogs aged 4 months or older). Veterinarians monitored the animals for up to 48 h after immunizations to verify the development of possible vaccine-related adverse effects. Throughout the study period (1-year follow-up and vaccination), the animals received no other vaccine or medication.

The 20 dogs naturally infected with *L. infantum* were from the CVL endemic area of Barra Mansa (Rio de Janeiro, Brazil). They presented positive results in the DPP^®^ screening test and in the EIE-leishmaniasis (Biomanguinhos/Fiocurz, Brazil) (EIE-CL) ELISA kit. Ten dogs were symptomatic to CVL and the other 10 were completely asymptomatic. Parasitological tests were positive in all dogs from the symptomatic group and in 9 out of 10 animals from the asymptomatic group (Table 1).

Clinical evaluations were performed by veterinarians and animals were classified according to a clinical score adapted from previous work (24, 25). Six common clinical signs (alopecia dermatitis, onychogryphosis, keratoconjunctivitis, the body condition, and lymphadenopathy) were scored on a semiquantitative scale, ranging from 0 (for absence of clinical signs) to 3 (severe). The sum of the values was used to determine the final clinical classification as low (0–2), moderate (3–6), or high (7–18). Ninety percent of the animals in the asymptomatic group were totally asymptomatic (score 0) and only 1 animal presented onychogryphosis (score 1). Inversely, 8 out of 10 symptomatic dogs (80%) presented high clinical classification, and the remaining two dogs were clinically classified as moderate (score 4) and low (score 1) (Table 1).

### Clinical Samples

Serum samples were obtained after blood collection and stored at −80°C for IgG, IgG1, and IgG2 ELISAs. Samples from controls (T0) and naturally infected dogs (CVL and ACVL) were also used for VL diagnosis with the DPP-LVC^®^ (Biomanguinhos^®^) screening test and the EIE-leishmaniasis ELISA kit (Biomanguinhos/Fiocurz, Brazil) (EIE-CL), all performed according to the manufacturer’s instructions.

In the control group (T0) and naturally infected dogs, bone marrow (BM) samples were also collected, according to the methodology of Abrantes et al. (26). Parasitological culture was performed according to the protocol of Ref. (27) and the quantitative polymerase chain reaction (qPCR) according to the protocol of Ref. (23) in the BM samples.

### Total Extract of *L. infantum* Promastigotes (LiAg)

Stationary phase culture promastigotes from *L. infantum* (MHOM/BR/1974/PP75) were submitted to 10 cycles of freezing and thawing (−196 and 37°C, respectively) and ultrasonication (40 W/15 min). Final protein concentration was adjusted to 1 mg/mL. Aliquots were stored at −80°C until the moment of use.

### *In Silico* Prediction of A2 Protein Linear B Cell Epitopes

Web server BepiPred was used for the prediction of linear B-cell epitopes (28). For each input FASTA sequence of *L. chagasi* A2 protein [GenBank: GQ290460], the server outputs an epitope prediction score for each amino acid. To determine potential B-cell linear epitopes, we utilized the recommended cutoff of 0.35, ensuring a specificity of 75% and sensibility of 49%. Linear B-cell epitopes of A2 protein were predicted to be located at the residues with the highest scores in at least nine consecutive amino acids. Sequences with BepiPred score above 0.35 considered potential linear B-cell epitopes.

Besides, Emini Surface Accessibility (ESA) was used to evaluate the probability of predicted linear B-cell epitopes to be exposed on the surface of the protein. This approach calculates the surface accessibility of hexapeptides and values greater than 1.0 indicate an increased probability of being found on the surface (29).

### Peptide Synthesis

Based on *in silico* prediction, all sequences with at least nine amino acids and Bepipred mean score above 0.35 were synthesized as a linear peptide by fluorenylmethoxycarbonyl solid-phase chemistry (30) (GenOne Biotechnologies, Brazil). Analytical chromatography of the peptides demonstrated a purity of >95% and mass spectrometric analysis also indicated an estimated mass corresponding to the mass of each peptide.

### ELISA for Specific IgG, IgG1, and IgG2 Isotypes

Levels of specific immunoglobulin IgG, IgG1, and IgG2 isotypes were measured by ELISA in the sera of infected dogs and healthy controls (before vaccination—T0), as well as 1 (T1), 6 (T6), and 12 months (T12) after immunization. Maxisorp 96-well plates (Nunc, Rochester, NY, USA) were sensitized with either LiAg, A2 protein (Leish-Tech^®^ vaccine Lot# 034/14), or each peptide (SV11, PP16, and VQ34) individually (1 μg/well) in PBS (phosphate-buffered saline) and conditioned overnight at 4°C. All ELISAs were performed using the reagents of Total IgG EIE-LEISHMANIOSE VISCERAL CANINA (Bio-manguinhos/Fiocruz, Brazil) following the manufacturer’s recommendations. Anti canine IgG1 and IgG2 peroxidase labeled antibodies were from Bethyl Laboratories Inc. (Montgomery, TX, USA) and were used in a 1:50,000 and 1:300,000 dilutions, respectively. Serum samples were tested in a 1:100 dilution. The absorbance was read at 450 nm using a Spectramax 250 ELISA reader (Molecular Devices, Sunnyvale, CA, USA).

Total IgG, IgG1, and IgG2 results were expressed as reactivity indexes (RI), calculated by the ratio of the mean optical density of tested samples by the mean optical density plus 1 SD of controls (T0). Dogs were scored positive to a particular antigen if the RI was higher than 1.

### Statistical Analysis

Wilcoxon’s paired non-parametric test was used to compare samples from healthy and immunized groups. The Mann–Whitney test was used to compare samples between vaccinated and CVL or healthy and CVL groups. Chi squared test for proportions analyses was performed to determine statistical differences between the frequencies of responders to each studied antigen in vaccinated and CVL groups. The results were considered significant when *p* ≤ 0.05 with a 95% confidence interval.

## Results

### Total IgG and Isotypes (IgG1 and IgG2) Levels Against A2 Protein and LiAg in Vaccinated and Naturally Infected Dogs

**Figure 1:**
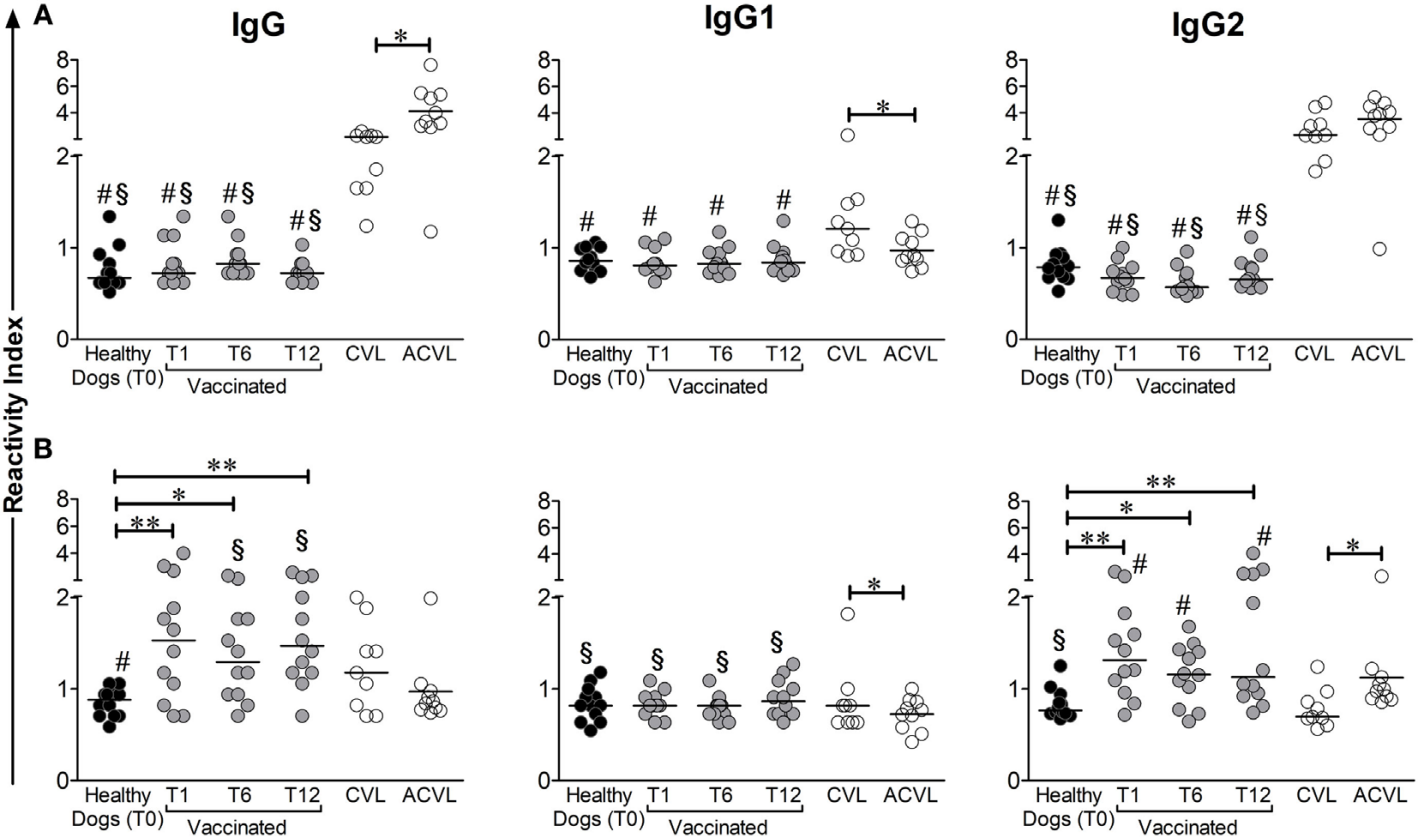
Medians of reactivity indexes of total IgG, IgG1, and IgG2 subclass against LiAg **(A)** and A2 protein **(B)** detected in the serum of infected symptomatic canine visceral leishmaniasis dogs (CVL), infected asymptomatic dogs (ACVL), healthy controls (T0), and vaccinated dogs 1 (T1), 6 (T6), and 12 months (T12) after the end of immunization protocol. Wilcoxon’s paired non-parametric test was used to compare samples from healthy and immunized groups. The Mann–Whitney test was used to compare samples between vaccinated and CVL or healthy and CVL groups. ^#^different from CVL, ^§^different from ACVL. *p < 0.05, **p < 0.005.

Dogs immunized with Leish-Tec^®^ showed varying titers of anti-A2 antibodies (RI ranging from 0.7 to 3.0). Total IgG levels increased after vaccination and remained elevated up to 12 months after the end of the vaccination protocol (*p* < 0.005). Animals with active CVL also showed positive titers of anti-A2 antibodies when compared to the control group (T0) (Figure 1B) (*p* = 0.048). Asymptomatic CVL animals, on the other hand, presented anti-A2 IgG titers similar to controls and significantly lower than vaccinated dogs on T6 (*p* = 0.0376) and T12 (*p* = 0.0051). The significantly higher RI of anti-A2 IgG antibodies observed in the vaccinated group was accompanied by a high IgG2 subclass RIs, whereas no change was observed for IgG1. It was also observed that, after vaccination, IgG2 levels were statistically higher in the vaccinated groups than in the CVL group (Wilcoxon *p* < 0.005). Interestingly, asymptomatic dogs showed the lowest magnitude of anti-A2 IgG1 levels and produced significantly more anti-A2 IgG2 antibodies than controls (*p* = 0.0076) and symptomatic CVL dogs (*p* = 0.0101) (Figure 1B).

Even though 70% of the animals in the symptomatic CVL group presented positive results in the anti-A2 total IgG ELISA, the frequency of responders for IgG1 and IgG2 subclasses was low (about 10%) (Figures 1B and 4). In contrast, when we evaluated the antibody response specific for *L. infantum* total antigen (LiAg), the RIs obtained in naturally infected animals (both symptomatic and asymptomatic) were higher, and all of them presented positive results in total IgG ELISA. Although only 67% of symptomatic CVL animals showed positive RI for anti-LiAg IgG1-specific antibodies, these levels were higher than those observed in all other groups (Figures 1A and 4). Asymptomatic dogs presented the higher RIs in anti-LiAg IgG ELISAs among all tested groups, and anti-LiAg IgG2 RIs higher than vaccinated animals and controls (Figure 1A). In the vaccinated group, no differences were observed in total IgG and IgG1 or IgG2 levels during entire follow-up (T1, T6, and T12) compared to controls (T0) (Figure 1A).

### *In Silico* Analysis of A2 Protein and Identification of Three Potential Linear B-Cell Epitopes

**Figure 2:**
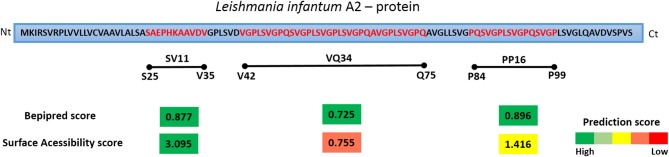
Schematic diagram of *Leishmania infantum* A2 protein and the predictions scores for linear B cell epitopes and surface accessibility. The regions corresponding to the residues 25–35, 49–64, and 67–95 of *L. infantum* A2 protein were selected for the synthesis of a soluble peptide based on the best prediction scores determined for both features. The prediction scores represents the average of scores for all amino acids within the region with prediction values above the cut-offs chosen for significance. The bar colors represent the intensity of prediction scores found, from green (high score) to red (low score).

In order to detect potential linear B-cell epitopes with surface accessibility for antibody recognition, the full sequence of A2 was analyzed using the BepiPred and ESA score. As shown in Figure 2, three high scored potential linear epitopes with at least nine amino acids were identified on the entire protein sequence: SV11 (aa 25–35 SAEPHKAAVDV), PP16 (aa 84–99 PQSVGPLSVGPQSVGP), and VQ34 (aa 42–75 VGPLSVGPQSVGPLSVGPLSVGPQAVGPLSVGPQ). The prediction scores showed similar values, ranging from 0.725 to 0.877. However, regarding the surface accessibility, SV11 presented the highest score (3.095), PP16 a moderate score (1.416), and VQ34 the lowest (0.755). Therefore, the predicted sequences were selected for further confirmation as a B cell epitope using a synthetic peptide and acquired antibodies of healthy, vaccinated, or infected (symptomatic and asymptomatic) dogs.

### Total IgG and Isotypes (IgG1 and IgG2) Levels Against A2 Protein Peptides in Vaccinated and Naturally Infected Dogs

**Figure 3:**
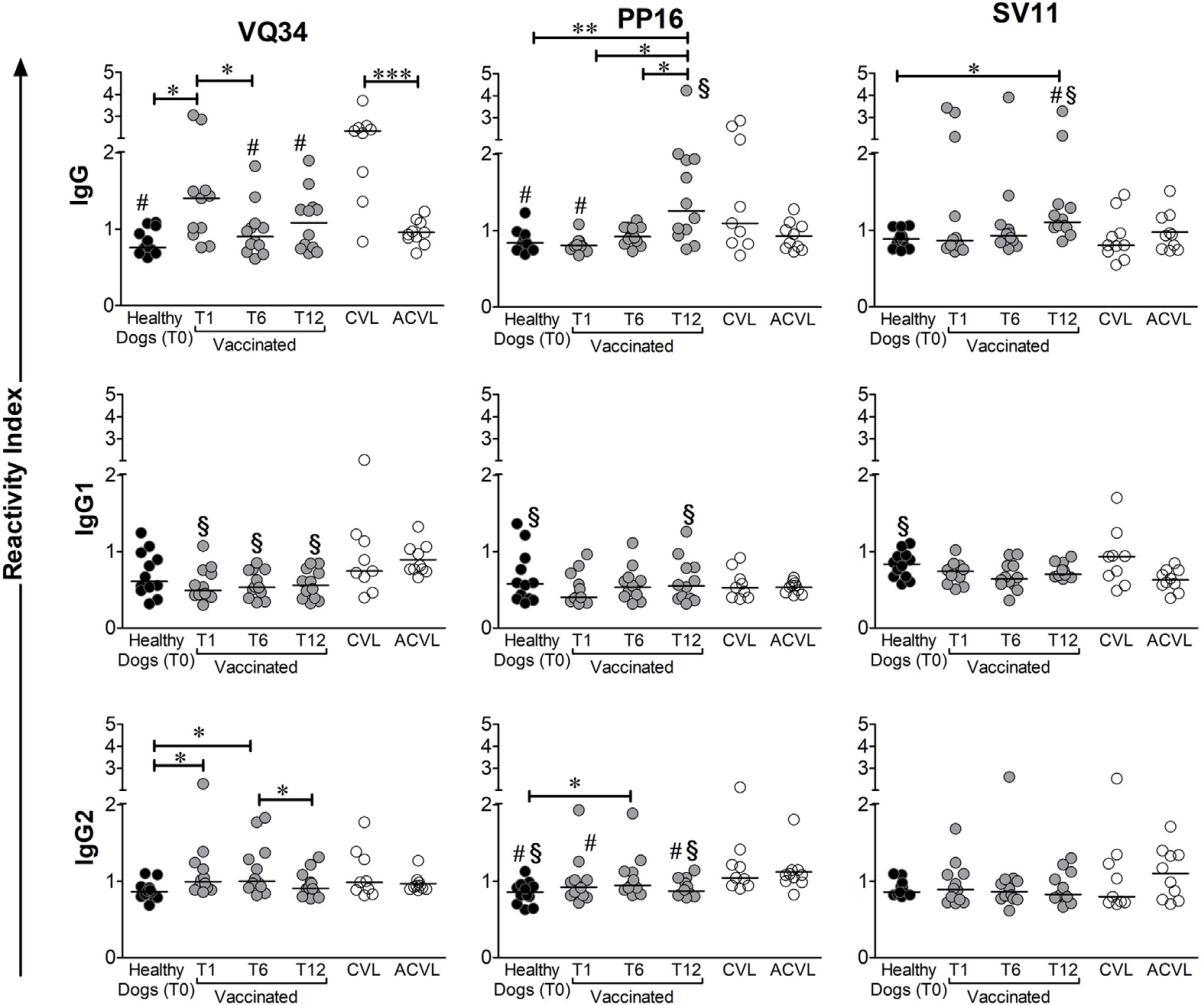
Medians of reactivity indexes of total IgG, IgG1, and IgG2 subclass against the three selected B peptides form A2 protein (VQ34, PP16, and SV11) detected in the serum of infected symptomatic canine visceral leishmaniasis dogs (CVL), infected asymptomatic dogs (ACVL), healthy controls (T0), and vaccinated dogs 1 (T1), 6 (T6) and 12 months (T12) after the end of immunization protocol. Wilcoxon’s paired non-parametric test was used to compare samples from healthy and immunized groups. The Mann–Whitney test was used to compare samples between vaccinated and CVL or healthy and CVL groups. ^#^different from CVL, ^§^different form ACVL. *p < 0.05, **p < 0.005, ***p < 0.0005.

Total IgG ELISAs demonstrated distinct patterns of response between peptides VQ34, PP16, and SV11 in the immunized and naturally infected groups. Regarding VQ34 peptide, the antigenicity was confirmed and the IgG RI ranged from 0.62 to 3.1 among all studied animals and time points. However, symptomatic CVL-infected animals have high production of IgG antibodies specific for this peptide, with higher reactivity index than asymptomatic (ACVL), healthy (T0), and vaccinated groups (T6 and T12; Figure 3). In addition, IgG antibodies in vaccinated dogs, at day 30, presented significantly higher levels when compared to healthy dogs (MW *p* = 0.0046). The levels of anti-PP16 also presented a wide spectrum of antibody reactivity, ranging from 0.67 to 4.2. The production of anti-PP16 IgG antibodies in the immunized group until the sixth month after vaccination was similar to healthy controls; however, those levels increased 1 year after vaccination (*p* < 0.05; Figure 3) and were higher than those observed in the asymptomatic infected group (ACVL *p* = 0.0248). In relation to SV11 peptide, the antigenicity was also confirmed and the magnitude of IgG response ranged from 0.72 to 3.9. Interestingly, despite not statistically significant, the presence of lower anti-SV11 antibody levels was observed in both symptomatic and asymptomatic naturally infected groups. In addition, 1 year after immunization, vaccinated dogs presented significantly higher RI than symptomatic and asymptomatic infected dogs as well as than healthy controls (*p* = 0.021, *p* = 0.047, and *p* = 0.0008, respectively) (Figure 3).

As regards the IgG isotypes studied, low IgG1 RI were observed against all peptides independently of studied group. On the other hand, IgG2 specific to all three described epitopes were detected in immunized and infected dogs. Although no response pattern was observed in none of the experimental groups, IgG2 against VQ34 was significantly higher in dogs vaccinated for up to 6 months (T1 and T6) after vaccination when compared to controls (T0). No profile could be observed among the studied groups concerning PP16 and SV11 peptides.

### Frequency of Responders Against Peptides Suggests a Potential Serological Marker to Distinguish Between Vaccinated and Infected Dogs

In order to better understand the differences observed in the serological responses against the selected peptides, a qualitative evaluation of the antibody responses found in the serum of the vaccinated animals was performed. The results were then analyzed by the percentage of dogs with positive total IgG antibody RI (greater than 1) 1 year after immunization (T12), in comparison to the frequency of responders in healthy controls (T0) and naturally infected canine visceral leishmaniasis symptomatic (CVL) and asymptomatic (ACVL) dogs.

**Figure 4:**
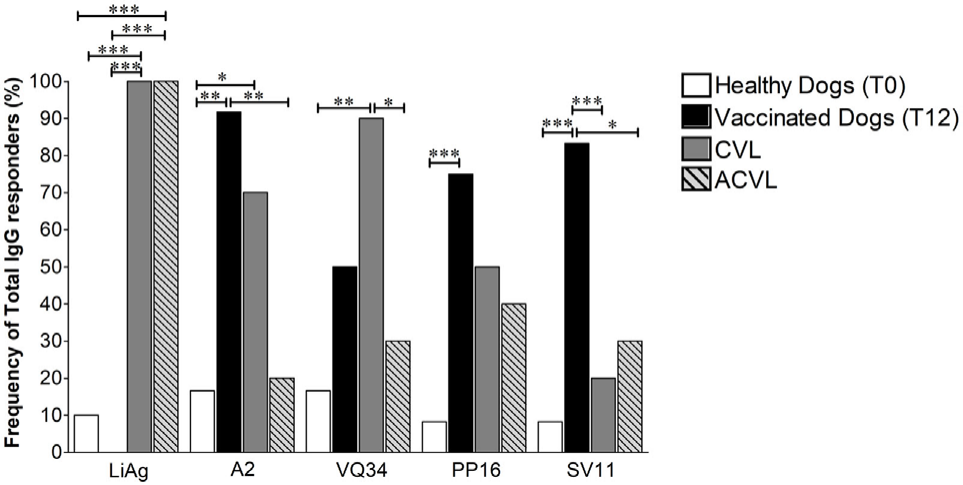
Frequency of total IgG responders observed in healthy controls (T0), vaccinated animals 1 year after immunization (T12) and in naturally infected canine visceral leishmaniasis symptomatic (CVL) and asymptomatic (ACVL) groups to LiAg, A2 protein (Leish-Tec^®^) and the three selected restricted B cell A2 protein peptides (VQ34, PP16, and SV11). The Chi squared test for proportions analyses was performed to determine statistical differences. *p < 0.05, **p < 0.005, and ***p < 0.0005.

As expected, all infected animals from the symptomatic (CVL) and asymptomatic (ACVL) naturally infected groups had positive responses to LiAg (100%) and both controls and vaccinated dogs groups showed significantly lower responses to LiAg when compared to CVL animals (Figure 4). On the other hand, both vaccinated (91.7%) and symptomatic CVL (70%) groups presented significantly higher percentage of positive reactivity to A2 protein when compared to health controls (16%) (*p* = 0.0006 and *p* = 0.0274, respectively). Vaccinated dogs also presented significantly higher frequencies of responders for anti-A2 IgG than the asymptomatic group (20% *p* = 0.0015).

Regarding the reactivity against the *in silico* selected peptides, although the percentages of responders in the vaccinated group were significantly higher in comparison to unimmunized healthy controls (T0) for all three peptides, they presented similar percentages when compared to infected symptomatic CVL group in relation to anti-VQ34 and anti-PP16 IgG responders. The frequency of animals with positive reactivity anti-VQ34 IgG was 90% in the CVL group and 70% in the vaccinated group (Figure 4). In contrast, only 30% of asymptomatic animals showed positive reactivity index for VQ34, a frequency very similar to that observed in controls (25%) and significantly lower than that observed in the symptomatic CVL group (*p* = 0.0198).

About 50% of the infected animals showed positive reactivity index for anti-PP16 IgG (40% ACVL and 50% CVL), while the frequency of responders in the vaccinated group was 80% (Figure 4). Interestingly, the frequency of responders in anti-SV11 IgG ELISAs reached high statically significant differences between not only vaccinated dogs (83.3%) and controls (8.3%) (*p* = 0.006) but also between vaccinated and naturally infected symptomatic (CVL—20%) and asymptomatic (ACVL—30%) groups (*p* = 0.0083 and *p* = 0.0274, respectively). The vaccinated animals that presented negative RIs on T12 (2 out of 12) had positive results both 1 month (T1) and 6 months (T6) after immunization.

## Discussion

The role of anti-leishmanial antibody response seen in VL is unclear. Hypergammaglobulinemia has long been recognized as a detrimental factor in VL, since higher antibody levels correlate positively with disease severity and decreases after cure (31–33). It was also demonstrated that most of the circulating IgGs are not parasite-specific, as a result of polyclonal B cell activation (34, 35).

Although there are a lot of evidences in the literature that support the view that *Leishmania*-specific antibodies are detrimental do the host (31, 36, 37), the role of antibodies and Fc receptors during *Leishmania* infections appears to be more complex, since the ligation of FcγRs on the surface of DCs and macrophages can lead to the induction of anti- or pro-inflammatory responses, depending on the IgG subclass, the cell type and/or the type of Fc receptors activated (38, 39). It was clearly demonstrated in cutaneous Leishmaniasis that parasite-specific IgG is required for efficient *L. major* uptake and IL-12 production by DCs (40), and that in *Leishmania*-coinfected mice, resolution of lesions required a specific antibody response, able to promote ROS production (41, 42).

It is also possible that antibodies may contribute to the protective response against *Leishmania* by neutralizing parasite virulence factors. Antibodies against the metalloprotease gp63 have been detected in the sera of VL patients (43), but it is not clear whether they are capable of neutralizing the parasites or has a protective function.

Dogs with CVL usually present greater titles of all IgG subclasses (44–46) and subclinical infection is usually associated with a lower antibody titers. Corroborating these data, we observed that infected dogs had higher levels of anti-LiAg antibodies than dogs vaccinated with Leish-Tec^®^, with predominance of IgG2 subclass. On the other hand, in our cohort, the asymptomatic dogs presented significant higher anti-LiAg IgG RIs than symptomatic animals. The most likely explanation for this finding is related with the fact that the majority of dogs in the symptomatic group had a high clinical classification, that is, they presented intense symptomatology, whereas 90% of the asymptomatic dogs did not presented any clinical symptoms. Suppression of the cellular specific immune response typically observed in chronic symptomatic dogs (47) could be involved with a deficiency in the production of B-cell specific antibodies in these animals, due to a decreased numbers of antigen-specific CD27+ memory B cells (35).

As opposed to the infected groups (CVL and ACVL), immunized dogs had baseline levels of anti-LiAg antibodies and in those that presented positive serology, although weakly reactive, the predominance was of IgG1. Similar results were reported by Iniesta et al. (48) indicating a predominance of anti-LiAg specific IgG2 antibodies in the serum of symptomatic CVL dogs and an increase in the production of anti-LiAg IgG1 antibodies in the serum of healthy dogs from non-endemic area. In the present study, we observed a predominance of LiAg-specific IgG2 antibodies in relation to IgG1 in both symptomatic and asymptomatic infected groups. Asymptomatic dogs also presented anti-LiAg-specific IgG1 antibodies levels similar to healthy controls and lower than the levels observed in the symptomatic group. Similarly, asymptomatic animals presented significantly higher titers of IgG2 and lower titers of IgG1 A2 protein-specific antibodies when compared to healthy controls and symptomatic animals. Although increased levels of specific LiAg IgG2 subclass in the sera of infected dogs were associated with protection (45), the evaluation of the IgG antibody class and its subclasses concerning signs of disease progression or protection, and the differentiation between vaccinated and infected animals, is still not well determined, since in the literature, the findings are controversial (45, 46, 48, 49, 50). It appears that the genetic heterogeneity of the dogs and the time of infection may influence antibody titers (46, 49, 51).

Despite the contrasting results available in the literature regarding the association of parasite-specific IgG subclasses with CLV susceptibility or protection, serological tests have been recommended for the CVL diagnosis due to the fact that they use less invasive methods of sample collection. However, they are still limited in the ability to distinguish between active and past or subclinical infection (52–54). Additionally, the antigens used in the serological tests, so far, may present cross reactivity with antibodies generated against other pathogens, leading to false positive results (11, 12, 55). In our cohort, an immunized dog seroconverted in the DPP-LVC^®^ 6 months after vaccination (T6), but the result were negative in the confirmatory tests (ELISA, qPCR, and parasitological culture). After 1 year, the DPP-LVC^®^ of this same dog was negative. As DPP-LVC^®^ uses different recombinant antigen than the vaccine, this positivity may be a result of a cross-reaction with other trypanosomatids (56), since, to date, none of studied dogs in the vaccinated group have been diagnosed with CVL.

Another hypothesis in endemic areas is that some dogs may develop a positive *Leishmania-*specific serology, but are not infected (6, 57). Thus, it is necessary to find new antigenic proteins, or even specific eptopes, which present higher sensitivity and specificity than the antigens used in the current kits.

Evaluating the antibody response specific to A2 protein, we observed that symptomatic infected animals and immunized animals presented higher titers of anti-A2 IgG antibodies than the health, unimmunized control group. Increased IgG levels were maintained in the immunized group up to 1 year after immunization (T12). The increase in total IgG was accompanied by an increase in the titers of anti-A2 IgG2 subclass in the vaccinated group. In addition, naturally infected symptomatic animals presented lower A2-specific IgG2 RIs than vaccinated animals at all study times (T1, T6, and T12) and similar to those observed in the healthy control group. On the other hand, asymptomatic CVL animals presented higher anti-A2 IgG2 levels than controls and symptomatic animals, suggesting an association between anti-A2 specific IgG2 subclass and protection. Similar results were described by Fernandes et al. (46), using beagle dogs, as well as in previous studies using animals of different breeds in areas of high endemicity (51, 56, 58).

Previous data in the literature have shown that hypergammaglobulinemia is established early after infection in VL and persist during the chronic phase, while the production of *Leishmania*-specific antibodies is short-lived (35). The authors correlated this non-specific hypergammaglobulinemia with a persistent expansion of a splenic B cell population with the atypical D21−CD27− phenotype. On the other hand, during acute VL, some B cells appear to be activated in a specific manner, and follow the follicular pathway where they interact with pre-Tfh cells. If these interactions are productive, both cell types proceed to form a germinal center where Tfh cells promote affinity maturation and the selection of the B cells clones with the highest affinity. Although Tfh cells were able to infiltrate the spleen during acute VL, they were mostly absent at the chronic phase, paralleling the decline in CD27+ memory B cells and specific IgG, suggesting an inability to maintain a sustained Tfh response during the chronic phase of infection. Based on these findings, it would be possible to assume that the antigen-specific antibody response mediated after vaccination would be different from that detected in dogs with symptomatic CVL. Indeed, regarding antigen-specific T cell responses, differences in the type of immune response triggered against parasite antigens in groups of vaccinated and naturally infected individuals have already been described in human cutaneous leishmaniasis (59–61).

Although some recombinant proteins have been evaluated as markers of disease (62, 63), a specific antigen able to differentiate healthy vaccinated animals from those with active CVL does not exist. Immunoinformatics emerges as an extremely interesting alternative in this context, since epitope prediction would lead to the selection and production of synthetic peptides with a greater possibility of recognition by the immune system, which are also, simpler, stable, and cheaper to produce (28, 64).

Therefore, when we evaluated the humoral response specific for A2 protein predicted linear B cell epitopes (SV11, PP16, VQ34), we observed differences in the total IgG responses according to the assessed peptide. One year after immunization (T12), vaccinated animals presented high serum-specific IgG to all three peptides. As expected for an antibody response elicited after immunization with recombinant proteins, the degree of positivity in the ELISA was associated with the surface accessibility score for each peptide (VQ34 < PP16 < SV11). This correlation was overturned in the symptomatic infected group, where the peptide with lower surface accessibility score (VQ34) presented the higher frequency of responders. On the other hand, asymptomatic animals presented low frequencies of responders to all three peptides (SV11—30%, PP16—40%, VQ34—30%).

The peptide VQ34 was highlighted as the one with the highest recognition by the serum of symptomatic dogs. Elevated levels of VQ34-specific IgG were detected in the serum of symptomatic dogs with a statistically significant difference compared to the other groups. Ninety percent (90%) of the animals in CVL group presented positive serology for VQ34 compared to 30% of the asymptomatic group. In a previous work of epitope mapping from A2 protein using the Spot-synthesis technique, Mendes et al. (65) described a high-reactivity B cell epitope in the serum of infected CVL animals, located within the repeating units PLSVGPQAVGLSVG (regions 67–81 and 122–135). Interestingly, the linear B-cell epitope VQ34 predicted *in silico* present these same repetitive units (region 67–95). We were additionally able to show that VQ34 is also recognized by the sera of vaccinated animals (50% of positivity), indicating its inability to discriminate vaccinated animals from those with active CVL.

Peptide SV11, on the other hand, showed high positivity in the vaccinated group, since 10 out of the 12 vaccinated dogs (83.3%) presented positive RIs for SV11 1 year after vaccination (T12). The remaining two animals showed borderline RIs at T12 (0.8 and 0.9) but presented positive RIs at both T1 and T6. We also observed a low positivity to SV11 in the serum of symptomatic (20%) and asymptomatic (30%) infected dogs, indicating that SV11 was able to better differentiate vaccinated animals from the naturally infected population.

In summary, we confirmed three main linear B-cell epitopes in A2 vaccine-based Leish-Tec^®^ that can be used as a peptide antigen. Moreover, the humoral response against SV11 peptide presented marked differences between infected and Leish-Tec^®^ vaccinated dogs, and the specific IgG response observed against SV11 should be further investigated, in large trials, to confirm its potential as a serological marker able to distinguish between infected and vaccinated dogs.

## Ethics Statement

This study was carried out in accordance with the recommendations of Conselho Nacional de Controle de Experimentação Animal—CONCEA, from the Brazilian Ministry of Science, Technology, Innovation, and Communications. The protocols were approved by the Ethical Committee for the Use of Experimental Animals (CEUA) of Fundação Oswaldo Cruz—FIOCRUZ (naturally infected animals—Protocol LW-4/17) and by Ethical Committee for the Use of Experimental Animals (CEUA) of Instituto Oswaldo Cruz—IOC/FIOCRUZ (healthy and vaccinated animals—Protocol L-45 / 2015).

## Author Contributions

Conceived and designed the experiments: JL-J and PL. Performed the experiments: MC, AR, and SS. Analyzed the data: MC, JL-J, PL, RR-D-S. Contributed reagents/materials/analysis tools: JL-J, FF, FM, PL, and SP. Wrote the paper: MC, PL, JL-J, and RR-D-S.

## Conflict of Interest Statement

The authors declare that the research was conducted in the absence of any commercial or financial relationships that could be construed as a potential conflict of interest.
