# Corrigendum: *Leishmania infantum* Virulence Factor A2 Protein: Linear B-Cell Epitope Mapping and Identification of Three Main Linear B-Cell Epitopes in Vaccinated and Naturally Infected Dogs

**DOI:** 10.3389/fimmu.2018.02245

**Published:** 2018-10-12

**Authors:** Monique Paiva Campos, Fabiano Borges Figueiredo, Fernanda Nazaré Morgado, Alinne Rangel dos Santos Renzetti, Sara Maria Marques de Souza, Sandro Antônio Pereira, Rodrigo Nunes Rodrigues-Da-Silva, Josué Da Costa Lima-Junior, Paula Mello De Luca

**Affiliations:** ^1^Laboratório de Pesquisa Clínica em Dermatozoonoses em Animais Domésticos, National Institute of Infectology Evandro Chagas-Fiocruz, Rio de Janeiro, Brazil; ^2^National Institute of Infectology Evandro Chagas-Fiocruz, Rio de Janeiro, Brazil; ^3^Instituto Carlos Chagas, Fundação Oswaldo Cruz, Curitiba, Brazil; ^4^Laboratório de Pesquisa em Leishmanioses, Instituto Oswaldo Cruz, Fundação Oswaldo Cruz, Rio de Janeiro, Brazil; ^5^Laboratório de Imunoparasitologia, Instituto Oswaldo Cruz, Fundação Oswaldo Cruz, Rio de Janeiro, Brazil

**Keywords:** canine visceral leishmaniasis, vaccines, A2 protein, serology test, epitope mapping, epitope prediction

In the original article, there was a mistake in the figure of Figure 2. Sequences of the peptides PP16 and VQ34 were placed in the wrong position in the schematic diagram of *L. infantum* A2 protein. In addition, the given initial and final amino acid residues from the sequences of peptides PP16 and VQ34 were incorrect in the figure legend. The corrected Figure 2, along with the corrected legend, appears below.

**Figure 2:**
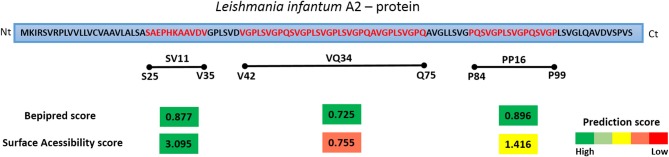
Schematic diagram of *L. infantum* A2 protein and the predictions scores for linear B cell epitopes and Surface Accessibility. The regions corresponding to the residues 25–35, 42–75, and 84–99 of *L. infantum* A2 protein were selected for the synthesis of a soluble peptide based on the best prediction scores determined for both features. The prediction scores represents the average of scores for all amino acids within the region with prediction values above the cut-offs chosen for significance. The bar colors represent the intensity of prediction scores found, from green (high score) to red (low score).

In the original article, there was an error in the abstract. The SV11 and PP16 peptide sequences were switched and the PP16 peptide sequence shown contained one additional amino acid. The correct sequence is PQSVGPLSVGPQSVGP. A correction has been made to Abstract. The correct text appears below.

In Brazil, canine visceral leishmaniasis (CVL) is caused by *Leishmania infantum*, presenting a broad spectrum of clinical manifestations. Dogs are the main parasite reservoir in urban areas and canine cases precede human infection. Currently, A2 protein based Leish-Tec® vaccine is the only vaccine commercially available against CVL in Brazil. Considering that the main screening and confirmatory tests of canine infection are serological, it is possible that the antibody response elicited after vaccination interfere with diagnosis, leading to the inability to distinguish between vaccinated and infected animals. In order to identify the specific B-cell response induced after vaccination, A2 protein sequence was screened for main linear B-cell epitopes using *in silico* prediction (Bepipred) and immunological confirmation by ELISA. Three amino acid sequences were described as potential B-cell epitopes (SV11-SAEPHKAAVDV, PP16-PQSVGPLSVGPQSVGP, and VQ34-VGPLSVGPQSVGPLSVGPLSVGPQAVGPLSVGPQ). Specific IgG ELISAs were performed in sera of 12 immunized dogs living in non-endemic areas, followed for up to 1 year after immunization. The results were compared with those obtained in a group of 10 symptomatic and 10 asymptomatic CVL dogs. All predicted epitopes were confirmed as linear B-cell epitopes broadly recognized by sera from studied dogs. Total IgG ELISAs demonstrated distinct patterns of response between peptides in the immunized and CVL groups. VQ34 peptide was recognized by the majority of sera from vaccinated and symptomatic dogs, and increases after vaccination. PP16 induced low levels of specific IgG that increased 1 year after immunization. Interestingly, a low frequency of reactivity was found against SV11 in naturally infected dogs (symptomatic and asymptomatic), while 83.3% of vaccinated dogs presented positive responses 1 year after immunization. The two animals in the vaccinated group that did not respond to SV11 1 year after immunization presented positive serology both 30 days and 6 months after immunization. In summary, we identified three main linear B-cell epitopes in A2 based vaccine. Moreover, the humoral response against SV11 presented marked differences between infected and Leish-Tec vaccinated dogs, and should be further investigated, in large trials, to confirm its potential as a serological marker able to distinguish between infected and vaccinated dogs.

In the original article, there was an error in the Results section. The given initial and final amino acid residues from the sequences of peptides PP16 and VQ34 were incorrect and the PP16 peptide sequence shown contained one additional amino acid. The correct sequence is PQSVGPLSVGPQSVGP. A correction has been made to Results, *In silico* analysis of A2 protein and identification of three potential linear B-cell epitopes, *Paragraph 1*. The correct text appears below.

In order to detect potential linear B-cell epitopes with surface accessibility for antibody recognition, the full sequence of A2 was analyzed using the BepiPred and ESA score. As shown in Figure 2, three high scored potential linear epitopes with at least nine amino acids were identified on the entire protein sequence: SV11 (aa 25–35 SAEPHKAAVDV), PP16 (aa 84–99 PQSVGPLSVGPQSVGP), and VQ34 (aa 42–75 VGPLSVGPQSVGPLSVGPLSVGPQAVGPLSVGPQ). The prediction scores showed similar values, ranging from 0.725 to 0.877. However, regarding the surface accessibility, SV11 presented the highest score (3.095), PP16 a moderate score (1.416), and VQ34 the lowest (0.755). Therefore, the predicted sequences were selected for further confirmation as a B cell epitope using a synthetic peptide and acquired antibodies of healthy, vaccinated, or infected (symptomatic and asymptomatic) dogs.

The authors apologize for these errors and state that this does not change the scientific conclusions of the article in any way.

The original article has been updated.

## Conflict of interest statement

The authors declare that the research was conducted in the absence of any commercial or financial relationships that could be construed as a potential conflict of interest.

